# Pigment Pattern in *jaguar/obelix* Zebrafish Is Caused by a Kir7.1 Mutation: Implications for the Regulation of Melanosome Movement

**DOI:** 10.1371/journal.pgen.0020197

**Published:** 2006-11-24

**Authors:** Motoko Iwashita, Masakatsu Watanabe, Masaru Ishii, Tim Chen, Stephen L Johnson, Yoshihisa Kurachi, Norihiro Okada, Shigeru Kondo

**Affiliations:** 1 RIKEN Center for Developmental Biology, Kobe, Japan; 2 Department of Biological Sciences, Graduate School of Bioscience and Biotechnology, Tokyo Institute of Technology, Yokohama, Japan; 3 Department of Pharmacology, Osaka University Graduate School of Medicine, Suita, Osaka, Japan; 4 Department of Genetics, Washington University School of Medicine, St. Louis, Missouri, United States of America; 5 Department of Biological Science, Graduate School of Science, Nagoya University, Nagoya, Japan; Stanford University School of Medicine, United States of America

## Abstract

Many animals have a variety of pigment patterns, even within a species, and these patterns may be one of the driving forces of speciation. Recent molecular genetic studies on zebrafish have revealed that interaction among pigment cells plays a key role in pattern formation, but the mechanism of pattern formation is unclear. The zebrafish *jaguar/obelix* mutant has broader stripes than wild-type fish. In this mutant, the development of pigment cells is normal but their distribution is altered, making these fish ideal for studying the process of pigment pattern formation. Here, we utilized a positional cloning method to determine that the *inwardly rectifying potassium channel 7.1 (Kir7.1)* gene is responsible for pigment cell distribution among *jaguar/obelix* mutant fish. Furthermore, in *jaguar/obelix* mutant alleles, we identified amino acid changes in the conserved region of Kir7.1, each of which affected K^+^ channel activity as demonstrated by patch-clamp experiments. Injection of a bacterial artificial chromosome containing the wild-type *Kir7.1* genomic sequence rescued the *jaguar/obelix* phenotype. From these results, we conclude that mutations in *Kir7.1* are responsible for *jaguar/obelix*. We also determined that the ion channel function defect of melanophores expressing mutant Kir7.1 altered the cellular response to external signals. We discovered that mutant melanophores cannot respond correctly to the melanosome dispersion signal derived from the sympathetic neuron and that melanosome aggregation is constitutively activated. In zebrafish and medaka, it is well known that melanosome aggregation and subsequent melanophore death increase when fish are kept under constant light conditions. These observations indicate that melanophores of *jaguar/obelix* mutant fish have a defect in the signaling pathway downstream of the α_2_-adrenoceptor. Taken together, our results suggest that the cellular defect of the Kir7.1 mutation is directly responsible for the pattern change in the *jaguar/obelix* mutant.

## Synopsis

Animals display a variety of skin pigment patterns. How these often intricate patterns are formed, however, is the longstanding question. Zebrafish is the only model organism having a pigment pattern, and thus it provides a unique system in which to investigate the mechanism of pattern formation. The striped pigment pattern of zebrafish comprises two types of pigment cells, melanophores (black chromatophores) and xanthophores (yellow chromatophores), and defects in pigment cell differentiation cause abnormal pigment patterns. However, the mechanism(s) underlying the arrangement of pigmented cells during development is unclear. In this paper, the authors cloned and studied the zebrafish mutant gene *jaguar/obelix* and identified it as *inwardly rectifying potassium channel 7.1* (*Kir7.1)*. Although the development of pigment cells is normal in *jaguar/obelix* fish, they have abnormally wide body stripes; thus, cell positioning is altered, suggesting that the *jaguar/obelix* functions in the system that determines pigment patterning. The connection between the Kir7.1 channel and the pigment pattern remains unclear, but the mutant melanophores are defective in intracellular aggregation and dispersion of the melanosome (pigment) controlled by the sympathetic neuron, suggesting that the signaling pathway activated by the neuron is also related to pigment pattern formation.

## Introduction

Many animals have fascinating color patterns on their skin, which have important roles in biological traits such as mate choice, camouflage, and the perception of threatening behavior [1,2]. How skin pigment patterns form, however, is a longstanding question among biologists. More than 50 y ago, a British mathematician, Alan Turing, proposed a theoretical hypothesis called the reaction-diffusion model [3], which explains autonomous pattern formation such as spots and stripes by the interaction and diffusion of hypothetical molecules [4–6]. The model has not been validated at the molecular level, however. Among model organisms used for genetic and/or developmental studies, zebrafish alone contains stripes on the skin, making it an ideal model for studying the molecular mechanism of pigment pattern formation. Zebrafish stripe patterns are determined mainly by the distribution of two types of pigment cells in the hypodermis during the larval-to-adult metamorphosis (approximately 2 wk)—melanophores (dark stripes) and xanthophores (light stripes) [7,8]. To understand how the stripe pattern forms, several studies have utilized zebrafish with skin pigment pattern mutations [9–12], and the molecular function of several genes responsible for the abnormal pigment pattern have been reported. In these studies, skin pigment pattern mutants were classified into two classes: fish with defects in the development of pigment cells comprised the first class, and fish with normal early stage development of pigment cells but disrupted adult stripe patterns comprised the second class. Investigation of mutant fish from the first class has revealed that the genes *mitf (nacre)* [13], *kit (sparse)* [14], and *ednrb1 (rose)* [15] are required for the development of melanophores and that *fms (panther)* [16,17] is required for the development of xanthophores. Interestingly, when one type of pigment cell does not develop normally, the other type fails to localize normally, suggesting that the interaction between different types of pigment cells plays a critical role in pigment pattern formation [8,9,18,19]. Two examples of the latter class of zebrafish mutants are *jaguar/obelix* [10,20] and *leopard* [20,21]. As described above, pigment cell development and distribution are normal in the early stage (approximately 2 wk, Figure S1) [19]. In addition, the adult fish display normal pigment cell development with altered spatial pigment patterning, suggesting that the underlying genes are required for pigment pattern formation [8,9,18,19].

Recently, we identified *connexin41.8* as the gene responsible for the *leopard* phenotype, and we determined that the gene product participates in both hemichannel and intercellular channel formation [22] (T. Chen and S. L. Johnson, personal communication). This finding suggests that small molecules such as ATP, cAMP, or IP3, which are transferred through these channels, may contribute to pigment pattern formation, although the molecular mechanism remains unclear. Further studies of genes from the second class of skin pigment pattern mutations will likely help to fully elucidate the molecular mechanism of pattern formation.

In this report, we show that the gene *inwardly rectifying potassium channel 7.1 (Kir7.1)* is responsible for the *jaguar/obelix* phenotype; thus, *Kir7.1* represents another pigment pattern mutant of the second class. We further demonstrate that the melanophores of *jaguar/obelix* mutants fail to aggregate or disperse melanosomes correctly in response to neural signals. In zebrafish and medaka, it has been reported that the constitutive stimulation of melanophores by light conditions or chemicals such as norepinephrine causes melanosome aggregation and subsequent death, resulting in pattern changes [23–25]. Here, we propose a possible explanation for the pigment pattern change in *jaguar/obelix* mutants by analyzing signal transduction defects with respect to melanophore response.

## Results

### Phenotypic Analysis of *jaguar* and *obelix* Mutants

**Figure 1:**
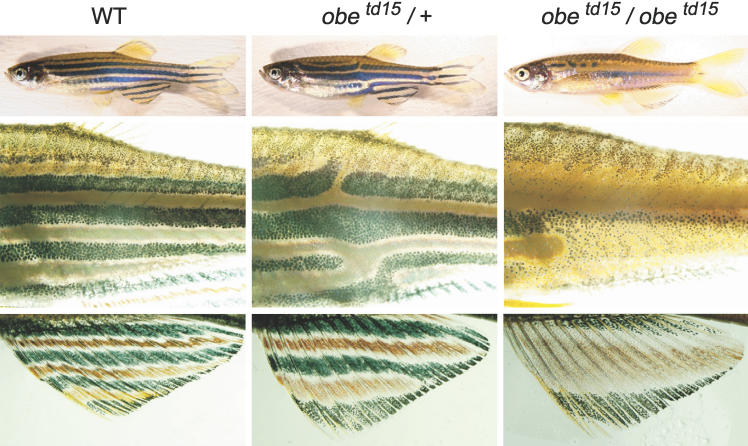
Adult Pigment Patterns in *jaguar/obelix* Mutant Zebrafish Pigment patterns of whole body (top), trunk (middle), and anal fin (bottom) in wild-type (WT), heterozygous (*obe^td15^*/+), and homozygous *(obe^td15^*/*obe^td15^)* fish. Pigment patterns in all alleles of *jaguar/obelix (jag^b230^, obe^tc271d^,* and *obe^td15^)* mutants are almost identical (unpublished data).

The *jaguar* mutant allele *jag^b230^* was previously identified as a spontaneous mutation [20], and the mutants of *obelix (obe^tc271d^* and *obe^td15^)* were previously identified by ENU mutagenesis [10,26]. All *jaguar* and *obelix* alleles have very similar phenotypes, and each of these mutations maps to the same locus on the zebrafish genome as determined by cross tests between *jaguar* and *obelix* fish (unpublished data). We called these mutant fish *jaguar/obelix*. Heterozygous mutants of *jaguar/obelix* have slightly fewer and broader stripes on the fins and some incomplete stripes on the body (Figure 1). Homozygous *obelix* mutants have broader yellow xanthophore bands on the fins and body and relatively thinner black melanophore stripes. Boundaries between melanophores and xanthophores are rather ambiguous in the homozygous mutant as compared to wild-type fish.

### Identification of the *jaguar/obelix* Gene

**Figure 2:**
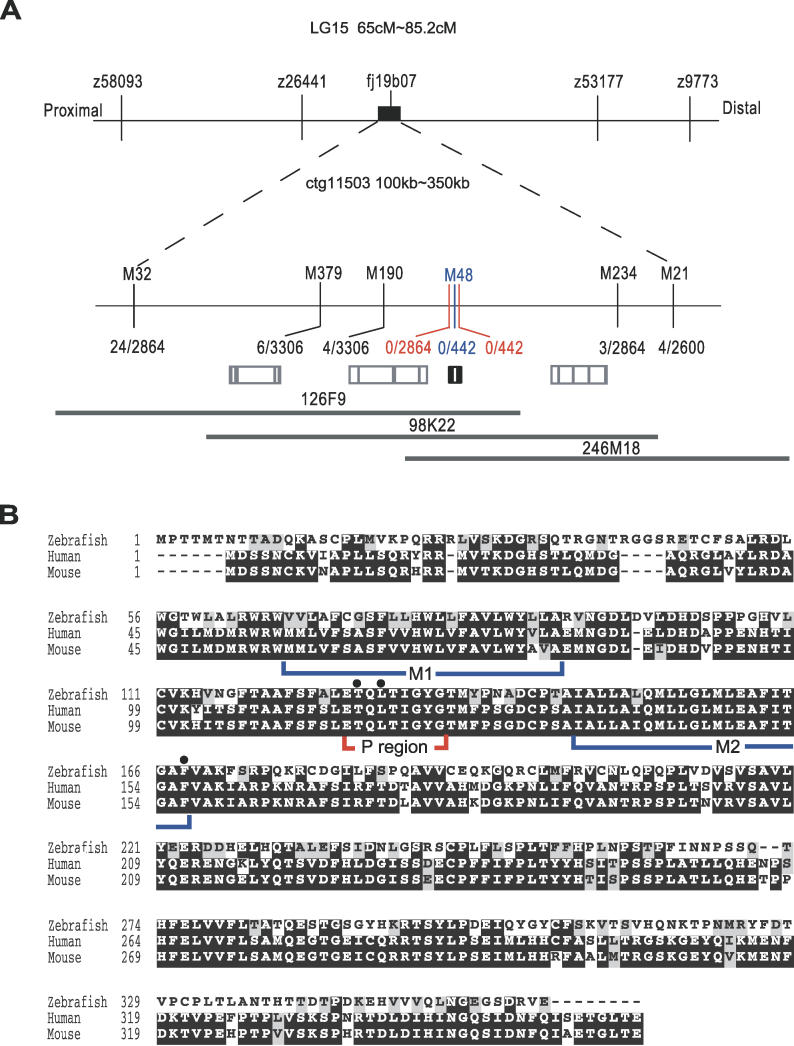
Positional Cloning and Sequence Alignment of the *jaguar/obelix* Gene (A) Line diagram depicting positional cloning of the *jaguar/obelix*. The *Kir7.1* locus is depicted by the small black box in the upper line; enlargement of the region bounded by the markers M32 and M21 is indicated by dashed lines. Open boxes with vertical lines represent the positions and intron-exon structures of putative genes predicted from the ENSEMBL transcript. Microsatellite markers and original markers are shown above the lines, and recombination rates are indicated below the lines. M48, which is located in the intron of *Kir7.1,* was perfectly linked to the mutant phenotype (blue vertical bar), and when we used the amino acid substitutions of Kir7.1 as the marker, they were also linked perfectly (red vertical bars). BAC clones (126F9, 98K22, and 246M18) covering this region are represented by gray lines. (B) Sequence alignment of Kir7.1 from zebrafish, human, and mouse. Positions of mutations identified in *jaguar/obelix* alleles are indicated by black circles. M1 and M2, transmembrane region; P, P-region (also shown in Figure 4A).

We used the *obe^tc271d^* and *obe^td15^* alleles of the *jaguar/obelix* mutant from the Tübingen background for positional cloning. From linkage analyses with microsatellite markers (Figure 2A), we mapped the *jaguar/obelix* locus between 73.2 cM (microsatellite marker z26441) and 79.6 cM (microsattelite marker z53177) on LG15. Fine linkage analysis with the polymorphic microsatellite markers mapped the *jaguar/obelix* locus between 0.12 cM (recombinant frequency 4/3,306) on the proximal side (M190) and 0.10 cM (recombinant frequency 3/2,864) on the distal side (M234). We found that marker M48 was perfectly linked to the mutant phenotype and was located in the intron of a gene homologous to mouse *inwardly rectifying potassium channel 7.1* (*Kir7.1*) (Figure 2B) [27]. In a critical region between M190 and M234, three genes, including *Kir7.1,* were predicted by ENSEMBLE transcript. We thus isolated cDNA sequences for *Kir7.1* and for two neighboring genes of the mutant alleles *(obe^tc271d^* and *obe^td15^)* and compared them with wild-type alleles. Both mutant alleles had nucleotide changes in *Kir7.1* that conferred amino acid substitutions, but no amino acid change was detected in either neighboring gene. We therefore concluded that zebrafish *Kir7.1* is a candidate gene for the *jaguar/obelix* mutant phenotype.

**Figure 3:**
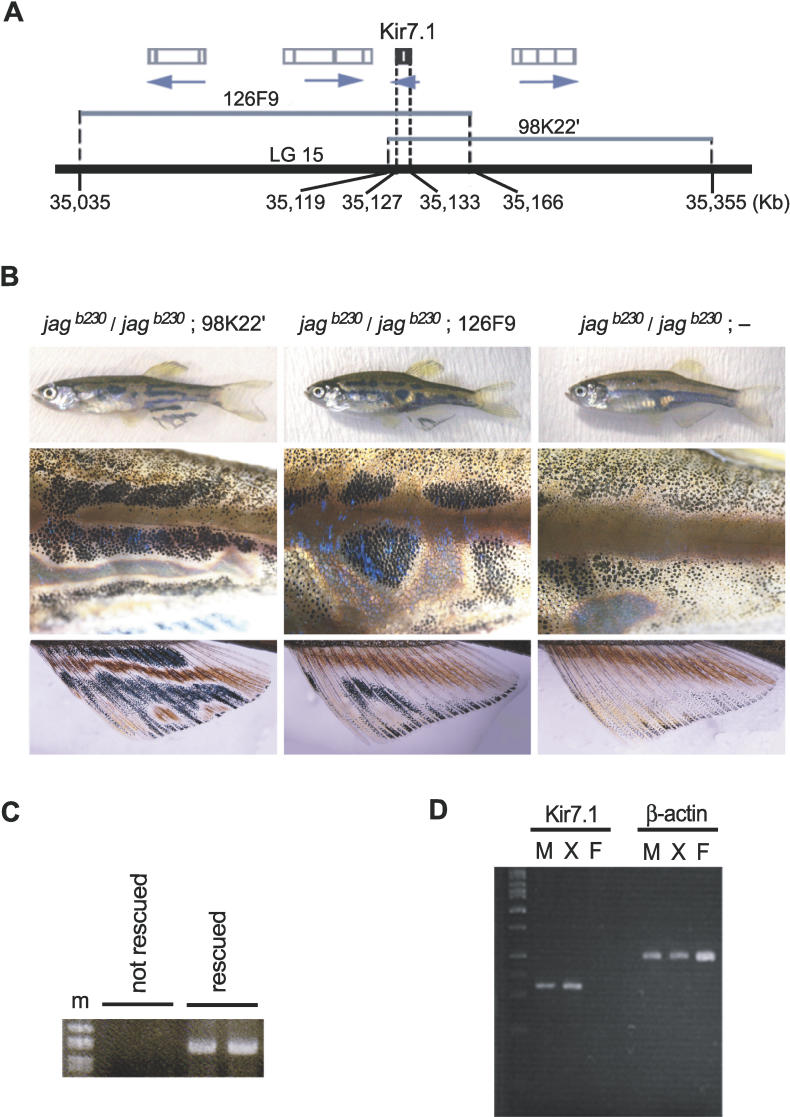
Rescue of the *jaguar/obelix* Phenotype by BAC Injection and Expression of *Kir7.1* mRNA (A) Line diagram depicting the genomic locations of the putative *Kir7.1* gene and BAC clones used for the rescue experiments. We used two BAC clones (98K22′ and 126F9, represented by gray lines) for microinjection. Numbers below the thick black line indicate the positions of the BACs and *Kir7.1* on LG15. Open boxes with vertical lines represent the positions and intron-exon structures of putative genes predicted from the ENSEMBL transcript. (B) BAC rescue of pigment pattern in zebrafish mutants. Fertilized eggs from homozygous mutant fish *(jag^b230^* and *obe^td15^)* were used in the phenotype rescue experiment. For the injected fish that survived to adulthood, representative pigment patterns of whole body (top), trunk (middle), and anal fin (bottom) are shown. The left panels depict patterns resulting from phenotype rescue using BAC clone 98K22′. The middle panels depict patterns resulting from phenotype rescue using BAC clone 126F9. The right panels depict patterns of noninjected fish. All mutant fish *(jag^b230^* and *obe^td15^)* rescued by BAC injection had a partial stripe patterns. (C) PCR analysis to confirm BAC integration in the rescued fin. Agarose gel analysis of PCR products derived from a BAC-specific sequence in nonrescued fins (lanes 2 and 3) and rescued fins (lanes 4 and 5). DNA fragments derived from the BAC clones were detected in all rescued fish (*n* = 6), but no PCR fragment was obtained from DNA of nonrescued fish (*n* = 6). Molecular mass markers are indicated (m). (D) Expression of *Kir7.1* mRNA as detected by single-cell RT-PCR. Agarose gel analysis of RT-PCR products derived from individual melanophore (M), xanthophore (X), or fin dermal cells (F). Molecular mass markers are indicated (m).

To confirm that we identified the correct gene for *jaguar/obelix,* we performed rescue experiments. Microinjections of mRNA to rescue the phenotype and/or injections of a morpholino-oligo to knockdown the wild-type phenotype were not effective for our mutant because stripe phenotypes of fish appear more than 1 mo after fertilization. In addition, initial attempts to rescue the *jaguar/obelix* phenotype by injecting DNA with constitutively activated *Kir7.1* promptly killed the embryos (unpublished data). We therefore performed microinjection of bacterial artificial chromosome (BAC) clones containing the genomic *Kir7.1* sequence (98K22′ and 126F9, see Figure 3A) into fertilized eggs. The injected BAC DNA should have become diluted as development proceeded; it is possible, however, that the BAC DNA was integrated into the chromosomes of some early embryonic cells, generating a chimeric fish with respect to the BAC DNA. For the injected fish that survived to adulthood, we obtained three mutant fish rescued by 98K22′ of 50 injected fish and three mutant fish rescued by 126F9 of 43 injected fish. None of the 100 injected fish that were produced with the BAC clone lacking *Kir7.1* showed a change of pattern. All mutant fish rescued by BAC injection had partial stripes with normal width or round spots of melanophores with a clear border between the xanthophore region on the fish body and fins (Figure 3B). The patterns rescued by BAC injection were quite similar to those produced in previous studies in which wild-type cells were transplanted into mutant eggs [19]. To further demonstrate that these pigment cell patterns resulted from the injected *Kir7.1* BAC clone, we performed PCR to detect the BAC-specific sequence in the rescued fin region of transgenic fish (Figure 3C). The BAC-specific sequence was detected in all fish fins that displayed the rescued pattern (*n* = 6), but it was not detected in fins that did not show the rescued pattern (*n* = 6). We concluded that *Kir7.1* is the gene responsible for the *jaguar/obelix* mutant phenotype.

### *Kir7.1* Is Expressed in Both Melanophores and Xanthophores

We analyzed *Kir7.1* mRNA expression in pigmented cells. Because the amount of mRNA expressed from the Kir family of genes in skin tissue is too low to detect via in situ hybridization or Northern hybridization (unpublished data), we performed RT-PCR on each type of pigment cell from adult tail fins (Figure 3D). We detected the *Kir7.*1 mRNA in both melanophore and xanthophore pigment cells but not in nonpigmented fibroblast cells from fish fin.

### Mutant Kir7.1 Malfunctions as an Inwardly Rectifying K^+^ Channel

**Figure 4:**
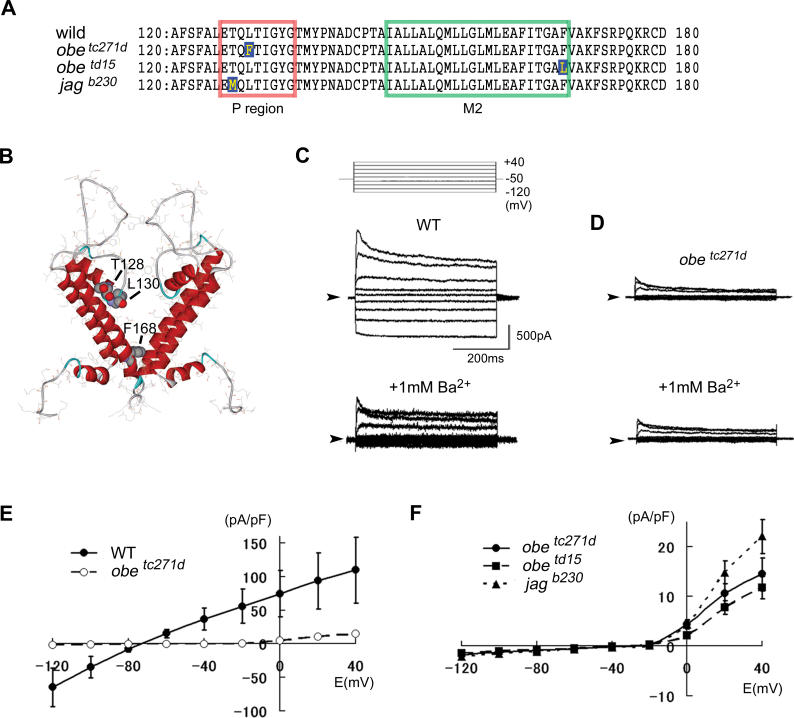
Functional Analysis of Kir7.1 Mutations (A) Positions of amino acid substitutions in the three alleles of *jaguar/obelix* with respect to wild-type. *obe^tc271d^* (L130F) and *jag^b230^* (T128M) have amino acid substitutions in the highly conserved P-domain (orange box), and *obe^td15^* (F168L) has mutations in the transmembrane region (green box). (B) Positions of the mutated amino acids in the 3D structure of zebrafish Kir7.1. The three-dimensional structure of Kir7.1 (residues 40 to 178) was deduced from the published X-ray crystal structure of KirBac1.1. Mutated residues (T128, L130, and F168) are highlighted as Corey-Pouling-Keltun (CPK) space-filling structures. (C–F) The electrophysiological properties of mutant zebrafish Kir7.1 expressed exogenously on HEK293 cell membranes. The voltage-clamp protocol and typical elicited currents on HEK293 cell membranes exogenously expressing zebrafish wild-type (WT) Kir7.1 are shown in (C). Elicited currents on HEK293 cell membranes for the mutant *obe^tc271d^* are shown in (D). Current inhibition in the presence of 1 mM Ba^2+^ is shown in (C) and (D), lower panel, indicating that the detected current is from potassium ions. Horizontal scale bar, 200 ms; vertical scale bar, 500 pA; arrowheads, zero-current levels. (E) Current (I)–voltage (V) relationship; wild-type (WT) (closed circles) and *obe^tc271d^* (open circles) (±SEM, *n* = 7, respectively). (F) Current (I)–voltage (V) relationship; *obe^tc271d^* (circles, ±SEM, *n* = 7), *obe^td15^* (squares, ±SEM, *n* = 7), and *jag^b230^* (triangles, ±SEM, *n* = 6). All mutant Kir7.1 show no functional current.

Kir7.1 belongs to an inwardly rectifying K^+^ (Kir) channel family**,** which is characterized by inwardly rectified K^+^ conductance. The Kir channel is a homo/heterotetramer of four Kir subunits that have two membrane-spanning helices (M1 and M2), a short helix, and a loop region (P). The G-Y/F-G motif in the P-region of this channel forms a filter for both the selection of K^+^ ions and their permeation across the channel [28]. The inner membrane portions of the four M2 helices, which cross one another, are thought to be crucial for channel gating [29]. Interestingly, we found that of the three *jaguar/obelix* mutants, two *(jag^b230^* and *obe^tc271d^)* contained amino acid substitutions in the P-region and one *(obe^td15^)* contained a substitution in the M2 helix (Figure 4A and 4B). To determine if these substitutions affected protein function, we performed voltage-clamp experiments to analyze HEK293 membranes exogenously expressing zebrafish Kir7.1 (Figure 4C to 4F). Wild-type Kir7.1 formed a functional channel on HEK293 cell membranes (Figure 4C and 4E). However, none of the mutant Kir7.1 channels we tested exhibited significant K^+^ conductance (Figure 4D–4F). Using FLAG-tagged constructs, we confirmed that surface expression of the channel was not affected by any of the mutations (unpublished data). We therefore concluded that all the mutations abolish K^+^ conductance by disrupting K^+^ permeation through the channel.

### Kir7.1 Mutation Leads to Dysfunctional Melanophore Aggregation and Dispersion Response

Although little is known about the function of Kir7.1 in mouse or human, some Kir channel genes are involved in a receptor-stimulated signaling cascade [30]. Therefore, we predicted that functional loss of Kir7.1 would cause defects in melanophore response to external signals. To address this hypothesis, we examined the aggregation and dispersion response of melanophores known to be controlled by various hormones and neurotransmitters [31–34].

**Figure 5:**
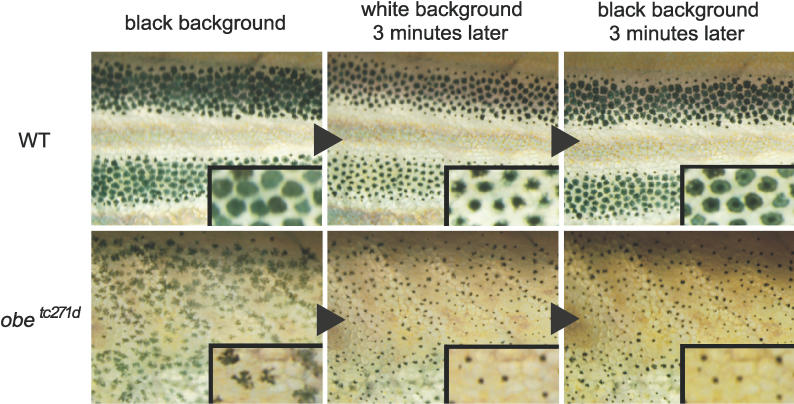
Melanophore Aggregation-Dispersion Response to the Change of Background Color Melanosome aggregation-dispersion in response to a rapid succession of background color change was measured. The upper panels show the responses of melanophores in the trunk of wild-type (WT) fish, and the lower panels show that of mutant fish *(obe^tc271d^)*. The left panels depict the usual state (black background), the middle panels depict melanophores after being sustained for 3 min in a white (light) background, and the right panels depict melanophores after 3 min in a black (dark) background. The wild-type melanophores responded normally to the change of background color (*n* = 6), whereas mutant fish *(jag^b230^* and *obe^tc271d^)* melanophores strongly responded to the change of background color from black to white but did not respond to the change from white to black (*n* = 9 for all).

Melanosomes typically aggregate in the center of melanophores when fish are placed in tanks with a white background, which leads to pale skin coloration. Conversely, when fish are placed in tanks with a black background, melanosomes disperse in dendrite form, and the skin color consequently becomes dark. These physiological responses are controlled by two systems. Neural signaling controls the quick response (approximately a few minutes) to background color change, and hormonal signaling controls the slow response (approximately hours) [31,35]. To determine the melanophore response of homozygous *jaguar/obelix* fish, we placed them in tanks with dark or light backgrounds for an extended period (1 d) and verified that the color adaptation of melanophores occurred normally (i.e., normal hormonal response; unpublished data). However, when the mutant fish were subjected to rapid alternation of background color (3-min intervals), melanophores from *jaguar/obelix* fish responded differently than those from wild-type fish (Figure 5, see Materials and Methods). The wild-type melanophores quickly responded to rapid background color changes in both directions (from dark to light, and from light to dark). Most of the melanophores in *jaguar/obelix* fish also responded to the rapid background change from dark to light, although the extent of aggregation in the mutant melanophores appeared to be more pronounced than in the wild-type. Interestingly, mutant melanophores did not disperse melanosomes in response to the rapid change of background from light to dark, whereas melanophores of the wild-type fish dispersed melanosomes quickly. We concluded that this mutant lacks a quick response system for melanophores. These observations suggest that the regulation of melanosome aggregation and dispersion, which is under neuronal control, is impaired in *jaguar/obelix* mutant fish.

### α_2_-Adrenoceptor Signaling Is Deficient in Melanophores of *jaguar/obelix* Mutant Fish

**Figure 6:**
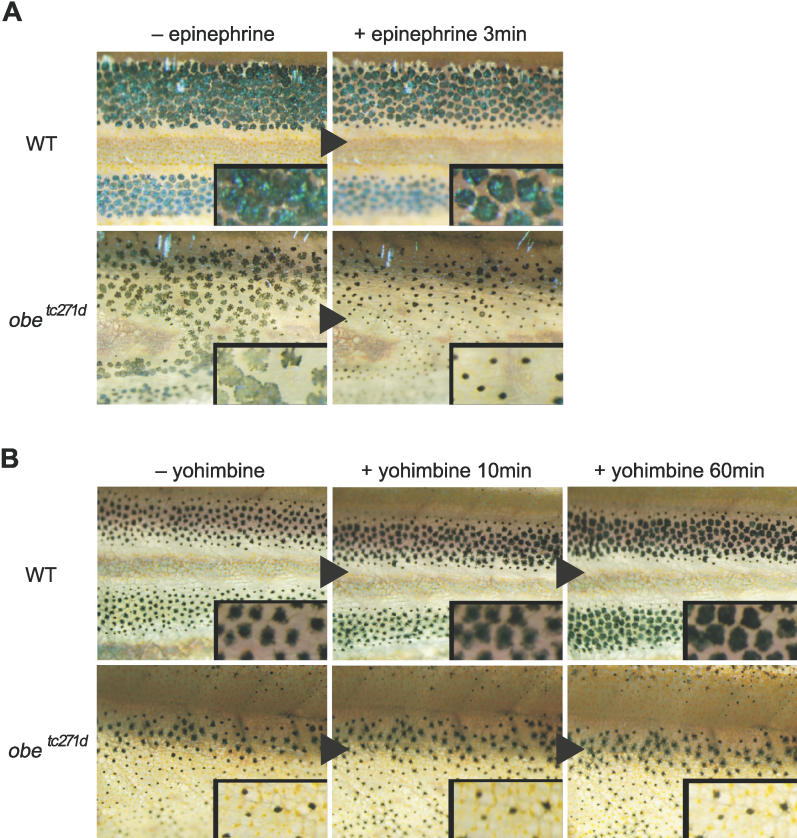
Melanophore Response to an α_2_-Adrenoceptor Agonist or Antagonist (A) Melanophore response to the agonist epinephrine. Fish were placed in black backgrounds for the duration of the experiment to block the endogenous aggregation signal from sympathetic nerves. The upper panels show the responses of melanophores in the trunk of wild-type fish (WT), and the lower panels show that of mutant fish *(obe^tc271d^).* Melanophores of wild-type (*n* = 6) and mutant fish *(jag^b230^* and *obe^tc271d^)* (*n* = 12, for all) showed melanosome aggregation when epinephrine was added in the breading water, although the extent of aggregation in the mutant melanophores appeared to be more pronounced than in the wild-type. (B) Melanophore response to the antagonist yohimbine. All fish were kept in an environment with a white background for several minutes (approximately 10 min) prior to addition of antagonist (left panels) and for the duration of the experiment to block endogenous dispersion. The upper panels show the responses of melanophores in the trunk of wild-type (WT) fish, and the lower panels show that of mutant fish *(obe^tc271d^)*. Melanosomes of wild-type fish dispersed after adding the antagonist, because the antagonist blocks the endogenous aggregation signal (middle and right panels) (*n* = 7). However, in mutant fish *(jag^ b230^* and *obe ^tc271d^),* many of the melanophores remained aggregated for a long time (longer than 60 min) (*n* = 12 for all) (unpublished data).

Several studies have reported that melanosome aggregation-dispersion in teleost fish is mainly controlled through the α_ 2_-adrenoceptor expressed in melanophores [31,33,36–39]. To determine whether the *jaguar/obelix* defect also is controlled by the melanophore α_2_-adrenoceptor, we examined the response of melanophores to either epinephrine (an α_2_-adrenoceptor agonist) or yohimbine (an antagonist) (see Materials and Methods). When the fish were placed in water containing epinephrine, melanosome aggregation was observed in both wild-type and mutant fish, but the extent of aggregation in the mutant melanophores appeared to be more pronounced than in the wild-type (Figure 6A). On the other hand, when the fish were placed in water containing yohimbine, melanophores in the wild-type fish dispersed melanosomes regardless of the white background color because the antagonist blocks the endogenous aggregation signal, but the mutant melanophores failed to disperse melanosomes and aggregation persisted for a long period (Figure 6B). These observations suggest a defect in the signaling pathway downstream of the α_2_-adrenoceptor in melanophores of *jaguar/obelix* mutant fish.

## Discussion

We have shown that Kir7.1 dysfunction causes wider stripes in *jaguar/obelix* mutant fish. It is difficult, however, to directly connect the known function of Kir7.1 with the phenotypes induced by the *jaguar/obelix* mutations. Several studies have shown that the role of the Kir family channel is to maintain membrane potential and sustain ionic composition [40,41]. Therefore, it is quite likely that many kinds of cell–cell signaling pathways that may be related to the pattern formation are affected by the dysfunction of the protein. It also has been reported that Kir family proteins are involved in a receptor-stimulated signaling cascade [30], and we predicted that functional loss of Kir7.1 would cause defects in the cellular response to external signals. Accordingly, we determined that *jaguar/obelix* mutant melanophores elicit a normal response to hormonal signals but an abnormal response to the neuronal signals that control melanosome aggregation and dispersion. Further tests utilizing an α_2_-adrenoceptor agonist (epinephrine) and an antagonist (yohimbine) suggested that mutant melanophores have a defect in the signaling pathway downstream of the α_2_-adrenoceptor.

In the α_2_-adrenoceptor pathway, it is important to note that melanosome aggregation in melanophores is caused by the reduction of cAMP concentration through G proteins, depending on the amount of α_2_-adrenoceptor ligand that binds the G protein [31]. Interestingly, it has been reported that one of the major functions of Kir family proteins is to sustain K^+^ homeostasis by potassium ion buffering and uptake, and that the loss of function of Kir channels can induce an increase of intracellular Ca^2+^ concentration [40,42]. Moreover, an increase of intracellular Ca^2+^ has pleiotropic effects on the activity of intracellular factors such as calmodulin-dependent cyclic nucleotide phosphodiesterase, which becomes activated and leads to the reduction of cAMP concentration [43]. Therefore, if Kir7.1 is related to the α_2_-adrenoceptor signaling pathway, it might have a role in maintaining ionic composition for normal cAMP signaling. This hypothesis provides a link between the Kir7.1 K^+^ channel defect and the abnormal melanosome movement found in *jaguar/obelix* mutants.

It is difficult to explain how stripe width changes upon a defect in Kir7.1 function because most of the complex interactions among pigment cells are largely uncharacterized. However, recent studies with zebrafish pigment pattern mutants suggest that pattern formation is controlled by mutual interactions among melanophore and xanthophore pigment cells and that these cells control each other's development, migration, and death [17,19]. This process stabilizes the balance between the number and position of melanophores and xanthophores and leads to normal stripe patterning. Therefore, the lack of control in cell development, migration, and cell death can result in abnormal pattern formation. In zebrafish and medaka, the constitutive induction of melanosome aggregation by norepinephrine causes cell death mediated by attenuation of cAMP-PKA signaling [23–25]. Our experimental results using *jaguar/obelix* fish indicate that the aggregation response was heavy and that aggregation was sustained for a long period even after the α_2_-adrenoceptor was blocked by yohimbine. Furthermore, we found that the melanophore density in mutant fish was significantly lower than in the wild-type fish (Figure S2). Therefore, we predict that the melanophores in the mutant fish may become more sensitive to the cell death signal, resulting in an aberrant interaction between melanophores and xanthophores that leads to pattern change. Based on the above reasoning, the newly characterized cellular phenotype described in this study may be critical for determining the link between genetic mutation and pigment pattern alteration.

To determine that the α_2_-adrenoceptor signaling defect directly leads to the wide-stripe phenotype, the signaling pathway must be controlled artificially in vivo. However, the pigment pattern does not appear clearly until the adult stage, and therefore signaling pathway disruption would result in abnormal fish development. In our preliminary experiment in which fish were maintained in breeding water containing epinephrine at a low concentration that did not inhibit the normal growth of the fish, we found that the pigment pattern of the wild-type fish became similar to that of heterozygous *jaguar/obelix* mutant fish (unpublished data). Thus, increased α_2_-adrenoceptor pathway activity may lead to the wider stripe phenotype, although this theory will require further confirmation.

We recently reported that the gene *connexin41.8* is responsible for the mutant phenotype of *leopard,* which has a round spot pattern [22] (T. Chen and S. L. Johnson, personal communication). This gene encodes another channel that can form both hemichannels and intercellular channels. The function of Connexin41.8 in pigment pattern change is unknown, but it is intriguing to note that one of the common features of *leopard* and *jaguar/obelix* is that small molecules affect pattern change. Additionally, Kir7.1 and Connexin41.8 are both thought to contribute to cell–cell interactions between melanophores, between xanthophores, and/or between melanophores and xanthophores [8,9,18,19]. Future detailed analyses of these interactions—and how channel function affects them—will be critical to the elucidation of the molecular mechanisms of pattern formation.

## Materials and Methods

### Zebrafish stocks.

Stocks of *obe^tc271d^* and *obe^td15^* were obtained from the Tübingen Stock Centre (Tübingen, Germany). *jag^b230^* was obtained from the Johnson lab (St. Louis, Missouri, United States).

### Genetic mapping and positional cloning.

Mapping of the *jaguar/obelix* locus was performed as described [44]. Fish carrying either the *obe^tc271d^* or *obe^td15^* mutant on a Tu genetic background were crossed with wild-type fish containing an AB or India genotypic background. F1 heterozygotes were interbred, and a genetic map of their F2 offspring was created using microsatellite markers (http://zebrafish.mgh.harvard.edu/zebrafish/index.htm). We identified six new markers in ctg11503 (ASSEMBLY Zv2) (Sanger Institute, Cambridge, United Kingdom) (http://www.sanger.ac.uk/Projects/D_rerio) for use in further mapping: M21, M32, M48, M190, M234, and M379. The recombinant numbers for F2 offspring were derived from five different parents (four from *obe^tc271d^* and one from *obe^td15^*) and were summed to calculate the crossover frequency. Because some of the parents did not have polymorphic sequence with respect to particular markers, the number of specimens tested was not identical. For gene prediction, we used ENSEMBLE transcript (Sanger Institute).

cDNAs of *Kir7.1* and neighboring genes were isolated using RNA extracted from tail fins via RT-PCR. To obtain full-length *Kir7.1,* we used the following primers: 5′-ATGCCTACCACCATGACA-3′ and 5′-ACTTCTTCTACTCCACGC-3′.

### Microinjection of the BAC construct.

Preparation and microinjection of the BAC DNA was done as described [45]. Approximately 100 kb upstream of *Kir7.1* in the dkye-98K22 BAC construct was deleted using a Counter-selection BAC Modification kit (Gene Bridges, http://www.genebridges.com), yielding the modified BAC clone 98K22′. The two BAC constructs, 98K22′ and dkey-126F9, were purified using the Qiagen Large-Construct kit (Qiagen, http://www.qiagen.com). The BAC DNAs (2 ng) were injected into fertilized eggs of homozygote fish *(jag^b230^* and *obe^td15^)* at the one- or two-cell stage. The injected eggs were maintained under normal breeding conditions as described [45]. BAC integration into chromosomes was confirmed by PCR amplification of a BAC-specific region. Primer sequences were 5′-TTGCAACAATCTCTCAGACG-3′ and 5′-ATCAATGGTTCAGGCTTGT-3′ for 98K22′ and 5′-TGTGAGTCTGATGCTCGTT-3′ and 5′-GAT TTAGGTGACACTATAG-3′ for 126F9.

### Single-cell RT-PCR.

Pigment cells in the tail fins were dissociated with collagenase type III as previously described [46]. Cells were dispersed in culture dishes, and then single melanophore, xanthophore, or dermal cells were isolated using glass capillaries. After genomic DNA was removed, cDNAs were generated by the SuperScript III CellsDirect cDNA Synthesis System (Invitrogen, http://www.invitrogen.com). The cDNA obtained from a single cell was dissolved in 30 μl of H_2_O, and a 5-μl aliquot was analyzed using RT-PCR to detect *Kir7.1* mRNA. As a control, 1 μl was analyzed to detect *β-actin* mRNA. PCR was carried out with primer sets that straddle an intron: 5′-CCTGGAGACGCAACTCACTA-3′ and 5′-TCGATGCTGAACTCCAGAGC-3′ for *Kir7.1* and 5′-AGGGTTACGCTCTTCCCCATGCCATC-3′ and 5′-GCGCTCAGGGGGAGCAATGATCT-3′ for *β-actin*. PCR amplifications were performed for 45 cycles for *Kir7.1* and 30 cycles for *β-actin* at 95 °C for 30 s, at 50 °C for 30 s, and 72 °C for 30 s, followed by 72 °C for 2 min.

### Molecular modeling.

The structure of the prokaryote inward rectifier K^+^ channel, KirBac1.1, was retrieved from the Protein Data Bank (PDB 1P7B) [29]. The pore structure homology model of the zebrafish Kir7.1 channel (residues 40 to 178) was created from the template KirBac1.1 structure using DS modeling software version 1.1 (Accelrys, http://www.accelrys.com). The quality of the models was assessed by their stereochemical properties, and root mean standard deviations were calculated by the software.

### Transient expression of zebrafish Kir7.1 on HEK293 cell membranes.

Plasmid construction and transfection into HEK293 cells were performed as described [47]. The coding regions of wild-type and mutant zebrafish *Kir7.1* were subcloned into the expression vector pcDNA3 (Invitrogen). Cotransfection of the *Kir7.1* plasmid with pCA-GFP, a plasmid expressing GFP, into the embryonic kidney cell line HEK293 was performed using LipofectAMINE PLUS (Life Technologies, http://www.invitrogen.com). Cells expressing GFP (and also possibly zebrafish Kir7.1) were identified by fluorescence microscopy and used for electrophysiology.

### Electrophysiological measurements.

Electrophysiological measurements were carried out as described [47]. For whole-cell recordings, the pipette (internal) solution contained: 150 mM KCl, 5 mM EGTA, 2 mM MgCl_2_, 3 mM K_2_ATP, 0.1 mM Na_2_GTP, and 5 mM HEPES-KOH (pH 7.3). The (external) bathing solution (normal Tyrode's solution) contained 115 mM NaCl, 20 mM KCl, 1.8 mM CaCl_2_, 0.53 mM MgCl_2_, 5.5 mM glucose, and 5.5 mM HEPES-NaOH (pH 7.4). Currents were recorded using the whole-cell configuration of the patch-clamp technique [48]. The tips of patch electrodes were coated with Sylgard (Dow Corning, http://www.dowcorning.com) and fire-polished. The tip resistance was 4 to 5 MΩ when filled with the pipette solution. All recordings were made at a holding potential of 50 mV. All experiments were performed at room temperature (approximately 25 °C). The channel current was recorded using a patch-clamp amplifier (Axon 200B; Axon Instruments, http://www.axon.com), low-pass-filtered at 1 kHz (−3 dB) by an eight-pole Bessel filter, digitized by an AD converter (Digidata; Axon Instruments), and continuously acquired on a computer (Dell) with commercially available software (pCLAMP9; Axon Instruments). Results are presented as mean values, and error bars represent ±SEM.

### Measurement of melanophore aggregation and dispersion response against background color.

Some of the older mutant fish (older than 5 mo) had a wild-type response to background color, particularly among females, and thus we used young adult fish (approximately 5 mo, around 2.5 cm long) to measure melanosome aggregation and dispersion against background color. Prior to analysis, the young fish were kept in the normal breeding environment (black background color) for at least 2 d to create uniform melanophore conditions for aggregation and dispersion measurements. After being photographed in black background conditions, the fish were transferred to a white cup for 3 min and then anesthetized by 0.02% tricaine (see [45]) and photographed. The fish were then transferred to a white cup without tricaine, and the awakened fish were transferred back to a black cup for 3 min, anesthetized, and photographed again. All photographs were taken quickly (approximately 1 min) to avoid melanosome dispersion caused by the heat from the microscope light or stress.

### Measurement of melanophore response to α_2_-adrenoceptor agonist and antagonist.

To evaluate the aggregation response of the α_2_-adrenoceptor to epinephrine, endogenous aggregation via the visual nervous system was blocked by maintaining a black background color. Epinephrine (Sigma) was applied to the breeding tank at a final concentration of 1 mM and the aggregation response was assessed for 3 min.

Likewise, to measure the α_2_-adrenoceptor dispersion response to the antagonist yohimbine (Sigma, http://www.signaaldrich.com), a white background color was maintained to block endogenous dispersion. We observed and scanned the response of the melanophore to yohimbine for a longer period (60 min) as the aggregation state was already induced by endogenous neurotransmitters that bind to α_2_-adrenoceptor tightly and because antagonist (yohimbine) competes with these neurotransmitters to bind the receptor. Yohimbine was added to the breeding tank to a final concentration of 10 μM.

## Supporting Information

Figure S1 Pigment Patterns in *jaguar/obelix* Mutant Zebrafish Pigment patterns of wild-type (WT), heterozygous (*obe^tc271d^/+*), and homozygous *(obe^tc271d^/obe^tc271d^)* fish at embryonic (48 h), larval (11 d, 19 d), and adult (90 d) stages. Differences in patterns emerge when adult pigment patterns begin to form around 14 d. Note that the pigment patterns in all alleles of *jaguar/obelix* mutants are almost identical.
(1.8 MB PDF)

Figure S2 Melanophore Density from Dark Regions on the Skin of Wild-Type Fish and *jaguar/obelix* Fish Shown are square mean melanophore densities from the dark regions on the dorsal skin of wild-type fish (WT) (*n* = 12) and mutant fish *(jag^b230^* and *obe^tc271d^)* (*n* = 12, respectively). All tested fish were approximately 30 mm in length. The statistical significance of differences between each group was assessed by the one-sided *t*-test. ***p < 0.0001. NS, not significant. Error bars represent ±SEM.
(358 KB PDF)

### Accession Numbers

The INSDC (International Nucleotide Sequence Database Collaboration; http://www.insdc.org) accession number reported in this paper for *Kir7.1* is AB271018.

## Abbreviations

BAC: bacterial artificial chromosome
*Kir7.1*: *inwardly rectifying potassium channel 7.1*
