# Supplementary figures and images for: Pigment Pattern in jaguar/obelix Zebrafish Is Caused by a Kir7.1 Mutation: Implications for the Regulation of Melanosome Movement
Source: PLoS Genet. 2006 Nov 24;2(11):e197. doi: 10.1371/journal.pgen.0020197 (PMC1657052; doi:10.1371/journal.pgen.0020197)

WT

*obe<sup>tc271d</sup>/+*

*obe<sup>tc271d</sup>/obe<sup>tc271d</sup>*

48h

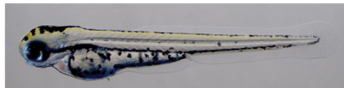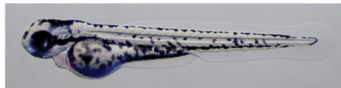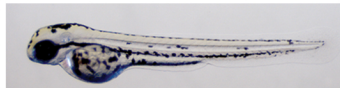

11d

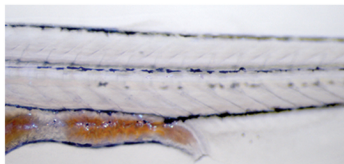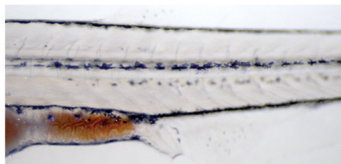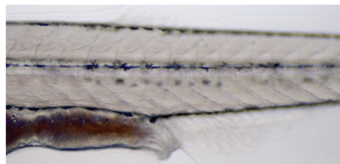

19d

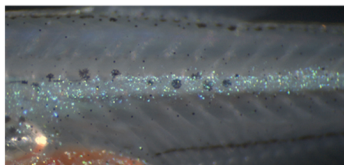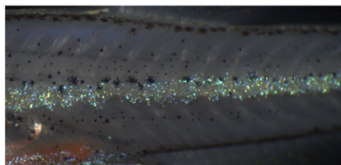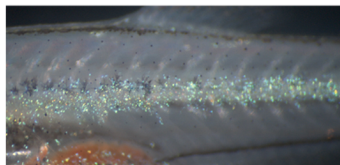

90d

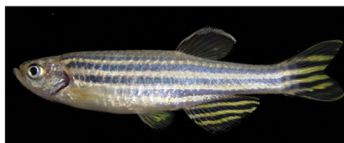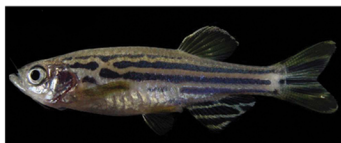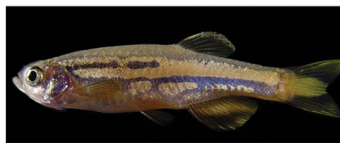

Supplement: Figure S1 — Pigment patterns of wild-type (WT), heterozygous (obetc271d/+), and homozygous (obetc271d/obetc271d) fish at embryonic (48 h), larval (11 d, 19 d), and adult (90 d) stages. Differences in patterns emerge when adult pigment patterns begin to form around 14 d. Note that the pigment patterns in all alleles of jaguar/obelix mutants are almost identical. (1.8 MB PDF) [file pgen.0020197.sg001.pdf]

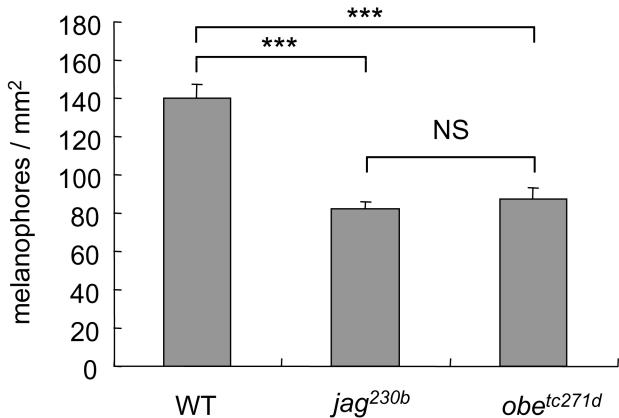

Supplement: Figure S2 — Shown are square mean melanophore densities from the dark regions on the dorsal skin of wild-type fish (WT) (n = 12) and mutant fish (jagb230 and obetc271d) (n = 12, respectively). All tested fish were approximately 30 mm in length. The statistical significance of differences between each group was assessed by the one-sided t-test. ***p < 0.0001. NS, not significant. Error bars represent ±SEM. (358 KB PDF) [file pgen.0020197.sg002.pdf]
